# Silexan in anxiety, depression, and related disorders: pharmacological background and clinical data

**DOI:** 10.1007/s00406-024-01923-8

**Published:** 2024-10-25

**Authors:** Siegfried Kasper, Anne Eckert

**Affiliations:** 1 Department of Molecular Neuroscience, Center of Brain Research, Medical University of Vienna, Spitalgasse 4, Vienna, A-1090 Austria; 2 Neurobiology Laboratory for Brain Aging and Mental Health, Psychiatric University Clinics Basel, Basel, Switzerland; 3 Research Platform Molecular and Cognitive Neurosciences, University of Basel, Basel, Switzerland

**Keywords:** Silexan, Lavender oil, Anxiety disorder, Depression, post-COVID-19, Review, Pharmacology

## Abstract

We present a narrative review of clinical trials investigating the anxiolytic and antidepressant effects of silexan, an active substance derived from lavender oil and summarize nonclinical findings from pharmacological studies supporting its therapeutic use. Six studies investigated the efficacy of the lavender oil in patients with subthreshold and generalized anxiety disorders as well as in mixed anxiety and depressive disorder (MADD). Furthermore, we present data indicating that silexan may influence sleep quality as well as anxiety or depressive disorders in individuals with post-COVID-19. Silexan taken orally at a daily dose of 80 mg for 10 weeks was significantly superior to placebo in reducing psychic and somatic symptoms of anxiety and was as effective as 0.5 mg/d lorazepam and 20 mg/d paroxetine. In patients with mild or moderate major depression, silexan was superior to placebo and comparably effective to 50 mg/d sertraline. Significant antidepressant effects were also observed in MADD and depression co-morbid with anxiety. The herbal product had a beneficial effect on activities of daily living and health-related quality of life. Adverse events associated with silexan in clinical trials were limited to eructation and mild, transient gastrointestinal complaints. The herbal product was not associated with drug interactions, sedation, sleep disturbance, dependence and abuse potential, sexual dysfunction, weight gain or withdrawal symptoms. Silexan was therefore safe and effective in subthreshold and syndromal anxiety disorders and in major depression.

## Introduction

Anxiety and depression are the most prevalent psychiatric conditions [1], collectively accounting for approximately half of all cases of mental disorders [2]. During the COVID-19 pandemic, the prevalence of anxiety and depression has increased further; with the consequences of this still being noticeable today [2].

In clinical practice, anxiety and depressive disorders are reported to be highly comorbid [3, 4]. According to an analysis of a German representative population sample, 59% of cases of generalized anxiety disorder (GAD) in a 12-months-period also met the diagnostic criteria for major depressive disorder (MDD). Of those diagnosed with MDD 10.8% also suffered from GAD and 44.6% from any anxiety disorder [5]. Co-occurring, clinical manifestations of anxiety and depression were found to be associated with greater severity and longer duration of illness, greater impairment, poorer clinical outcome, poorer response to treatment, and a higher risk of chronicity [4, 6, 7].

Anxiety and depression have been described as different manifestations of a vulnerability to a common ‘distress’ factor [8]. Moreover, distinct common neuroanatomical abnormalities were detected by neuroimaging in patients with anxiety and depressive disorders [9]. It is therefore not surprising that there is considerable overlap between anxiety and depressive disorders on the symptom level. The most important symptoms in both include restlessness, fatigue, impaired concentration and disturbed sleep [10, 11]. It has been discussed [12] that symptom overlap may be partly due to an artefact of the diagnostic systems since sleeping and concentration difficulties as well as fatigue and psychomotor agitation are among the key diagnostic criteria for both GAD and MDD according to the Diagnostic and Statistical Manual of Mental Disorders-5 (DSM 5) [13] and the ICD 11 [14]. However, anxiety and depression are obviously distinct emotional states. A distinguishing feature may be that the former tends to be more future-oriented and is characterized by worries about anticipated danger and harm whereas the latter is mainly past-oriented and thus characterized by rumination and self-deprecation [15].

A systematic review and meta-analysis of 66 studies involving more than 88,000 patients was performed on the prospective relationship between anxiety and depression [16]. It revealed that anxiety disorders were predictive of later depressive disorders (OR = 2.77) while depressive disorders were predictive of later anxiety disorders (OR = 2.73), with even closer associations over shorter time periods. The authors concluded that anxiety and depression bidirectionally and prospectively predict one another with similar strength [16]. In another study, the odds ratio (OR) for patients diagnosed with a syndromal anxiety disorder to develop MDD within the following year ranged between 7 for social phobia and 62 for GAD [17].

The diagnosis of GAD and MDD are strictly operationalised through diagnostic manuals, such as the DSM. Even patients with a significant burden of disease may not fulfil all criteria and may not receive proper treatment. For them, the terms subsyndromal, subthreshold or subclinical condition has been introduced. The most widely used definition for subsyndromal depression is a clinically relevant level of symptoms without meeting the diagnostic criteria for MDD in the DSM-system [18]. Although there is no general definition of subsyndromal GAD either, mostly the duration or the number of symptoms was reduced [19].

The interpretation that anxiety and depression have some pathopsychological mechanisms in common is supported by the fact that antidepressant drugs, including selective serotonin reuptake inhibitors (SSRIs) and selective norepinephrine reuptake inhibitors (SNRIs), were found to be efficacious and are recommended as first-line treatment in anxiety disorders [20]. Even though SSRIs and SNRIs have a more favourable safety profile than earlier-generation drugs such as benzodiazepines for anxiety and tricyclics for depression, they are still associated with partly disturbing side effects that may interfere with essential activities of daily living and may thus adversely affect the patients’ quality of life. Common side effects of SSRIs and SNRIs include anticholinergic effects, fatigue, sedation, somnolence as well as insomnia, sexual dysfunction, and weight gain or loss. Furthermore, possible drug interactions have to be considered [21], and a number of antidepressant or anxiolytic drugs have a well-documented potential for addiction and/or withdrawal effects [22–24].

Silexan^1^ is an essential oil produced from *Lavandula angustifolia* flowers that has been authorised as a medicinal product in more than 20 countries worldwide. The product is available in soft gelatine capsules with 80 mg of active substance for once-daily oral intake. We present a narrative review of the effects of silexan in the treatment of subthreshold and syndromal anxiety and depression based on a series of randomized clinical trials. Furthermore, we present data indicating that silexan may influence sleep quality as well as anxiety or depressive disorders in individuals with post-COVID-19. The mechanism of action is elucidated by findings from pharmacodynamic studies.

## Pharmacological background and nonclinical studies

**Fig. 1:**
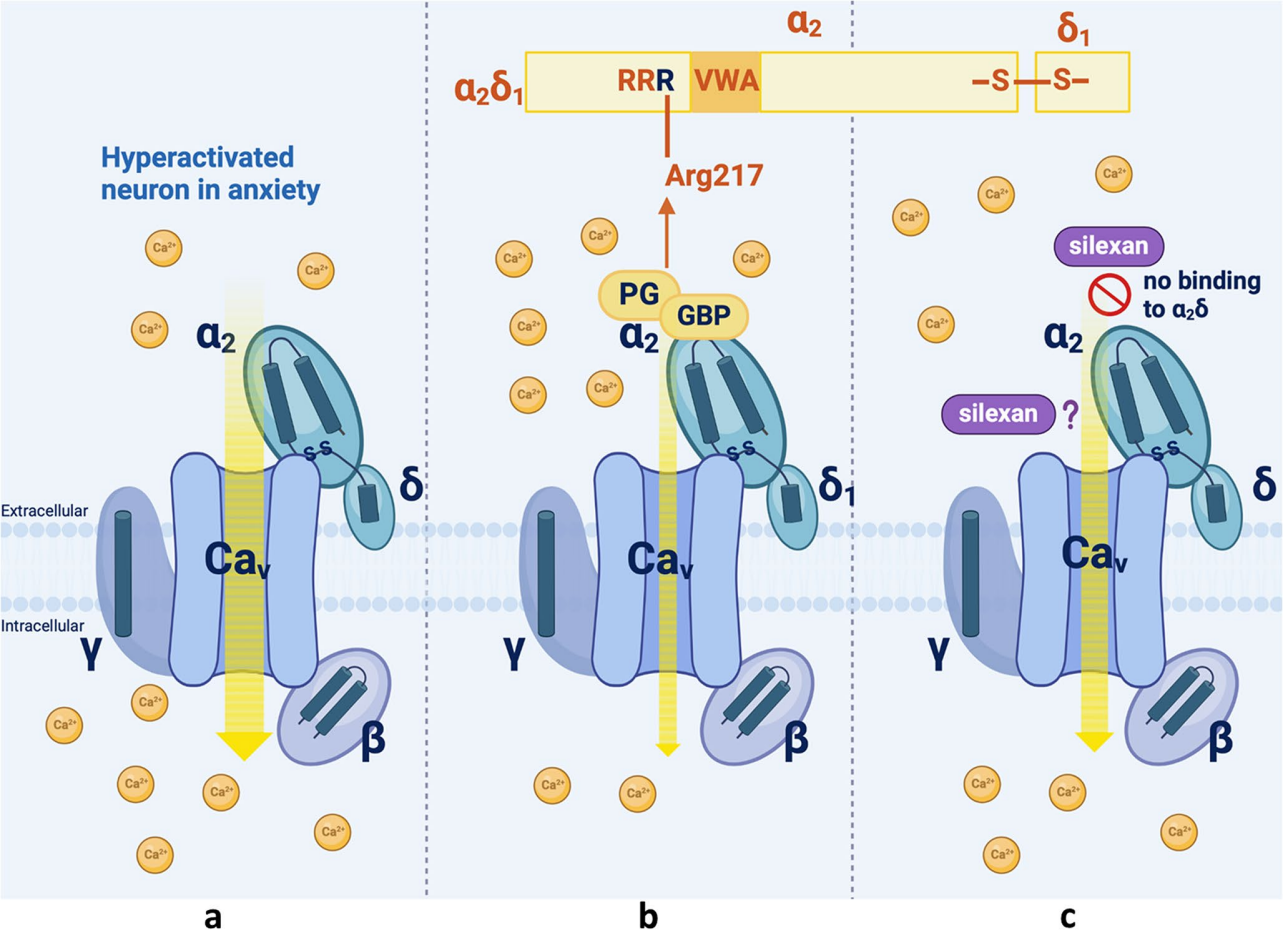
Mode of action of anxiolytic drugs on presynaptic voltage-gated calcium channels (VGCCs). Figure **a** – Schematic representation of a hyperactivated neuron exhibiting high calcium (Ca^2+^) influx at the presynaptic terminal. The calcium channel complex consists of the Cav pore-forming subunit and β, α2δ and γ ancillary subunits. Figure **b** – Mode of action of gabapentin (GBP) and pregabalin (PG). GBP and PG bind specifically to the α2δ1 subunit with high affinity, thereby not disrupting the association of α2δ1 to the Cav pore-forming unit, predominantly of Cav2.1 (P/Q-type). However, they do alter the properties of VGCCs by substantially reducing the magnitude of Ca^2+^. The order of the domains in α2δ1 is shown above the VGCC model. The RRR motif that is key to GBP / PG binding is situated on a loop just before the VWA domain. The third Arg in this RRR motif (highlighted in blue) termed Arg217 is critical for GBP / PG binding. Figure **c** - Mode of action of silexan. While silexan reduces calcium currents via VGCCs, potentially contributing to its anxiolytic effects, it does not specifically bind to the α2δ1 subunit (∅) as GBP / PG does. Moreover, it inhibits three VGCC subtypes (P/Q - Cav2.1, N - Cav2.2, T – Cav3). Silexan seems to have a moderate effect on VGCCs, showing a less pronounced reduction in calcium influx compared to GBP / PG (**b**). The precise mode of action of silexan on VGCCs is unknown. The figure was created with BioRender.com

**Fig. 2:**
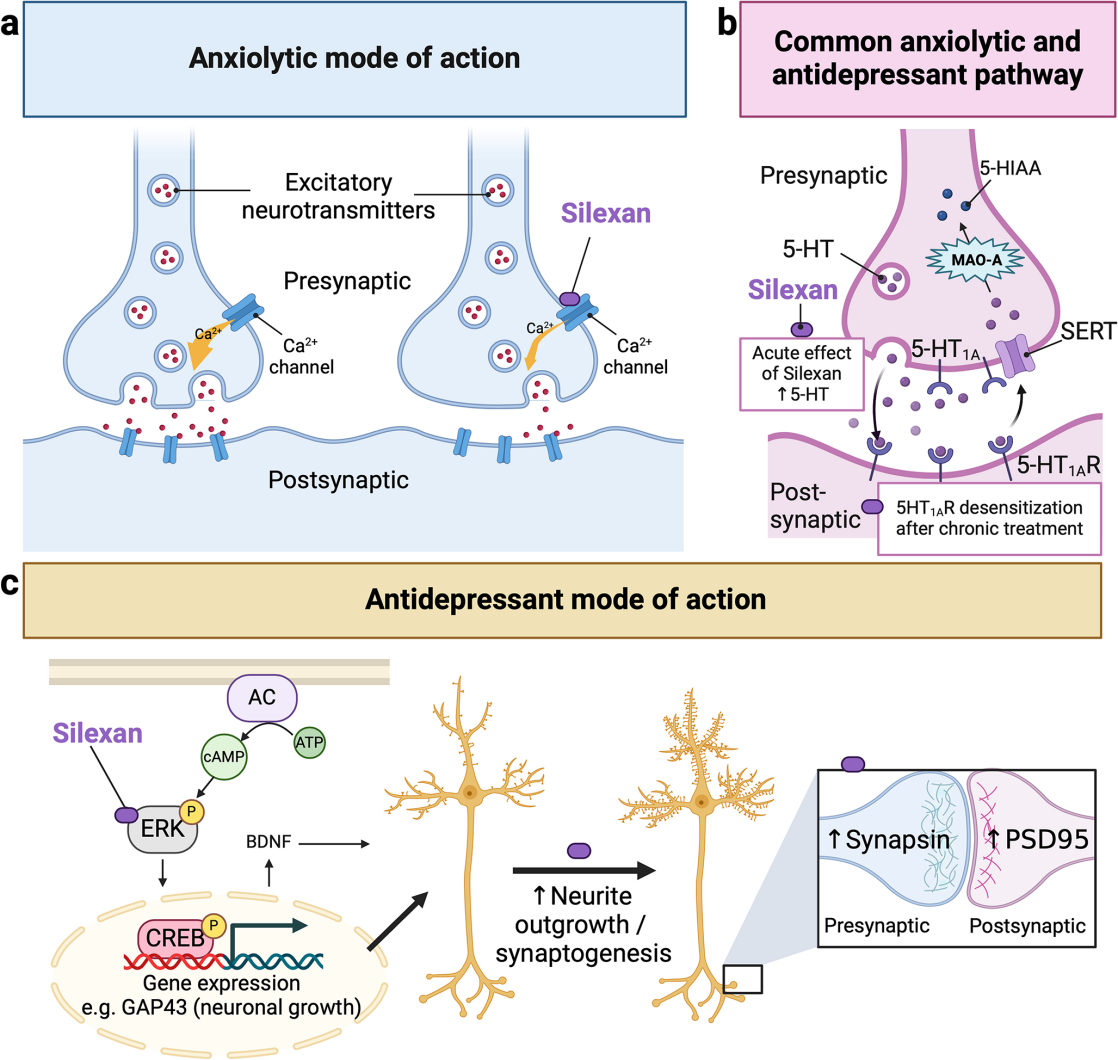
Potential modes of action of silexan on pathophysiological processes of anxiety and depression. Figure **a** - Anxiolytic mechanisms. Silexan moderately inhibits mainly T-type and N-type channels and to some extent P/Q-type channels. The VGCC inhibition decreases calcium influx into neurons upon activation of the channels at the presynaptic terminal. This leads to a reduced release of excitatory neurotransmitters, such as glutamate and substance P, into the synaptic cleft. Consequently, there is decreased neuronal excitability and more stabilized neuronal activity at the postsynaptic terminal. Figure **b** - Common anxiolytic and antidepressant pathway. Silexan has been shown to increase extracellular serotonin levels in the prefrontal cortex following a single *acute* administration; however, unlike SSRIs, this increase is not due to inhibiting the serotonin reuptake transporter (SERT). *Prolonged* treatment with silexan may induce similar adaptive changes to those observed with SSRIs, such as reduced 5-HT1A heteroreceptor binding at the postsynaptic terminal. These mechanisms may be involved in both affective and anxiety disorders. The exact mechanism by which silexan modulates the serotonergic system remains unclear. Figure **c** - Antidepressant mode of action. Silexan acts via the cAMP-PKA-CREB-BDNF pathway initiating CREB phosphorylation, promoting neurite outgrowth (reflected by an increase in neurite length) and enhancing synaptogenesis. The zoomed-in inset illustrates the elevated levels of pre- and postsynaptic proteins, specifically synapsin and PSD95, respectively. The figure was created with BioRender.com. *Abbreviation* 5-HIAA: 5-Hydroxyindoleacetic acid is the main metabolite of serotonin, 5-HT: serotonin, 5-HT1AR: a subtype of serotonin receptors, AC: adenylyl cyclase, ATP: adenosine triphosphate, BDNF: brain-derived neurotrophic factor, cAMP: cyclic adenosine monophosphate, CREB: cAMP response element-binding protein, ERK: extracellular signal-regulated kinase, GAP43: Growth-associated protein-43, PSD95: Postsynaptic density protein-95, SERT: serotonin transporter

In vitro and in vivo experiments have demonstrated that silexan possesses both anxiolytic and antidepressant activities (Table 1). Although its exact mode of action it is not completely understood to date, it obviously involves modulation of neurotransmitter systems and enhancement of neuroplasticity (Figs. 1 and 2).

**Table 1:** Activity of silexan in in vitro and in animal models indicative of anxiolytic and antidepressant properties

Test system	Conc./Dose	Time of treatment	Outcome measure/Effect of silexan	Reference
**Anxiolytic properties**				
**In vitro**				
*Rat* Primary hippocampal neurons *Mouse* Synaptosomes	0.01–30 µg/ml Active control: Pregabalin 0.03–100 µM	Acute (10 min)	Reduced Ca2+-transients after KCl-induced activation of VOCCs: ⇒ *Effective range*: *0.1–30 µg/ml*	[26]
*Transfected cell lines* each expressing only one VOCC subtype (CHO, HEK293 cells)	1 or 10 µg/ml	Acute (50 s)	Inhibition of N-, T- and P/Q-type VOCCs in whole cell patch-clamp experiments: ⇒ *Effective range*: *1–10 µg/ml*	
**In vivo**				
*Freely moving rats* Microdialysis probe into the medial prefrontal cortex	3,10 or 30 mg/kg i.p.	7 days	Increased extracellular levels of noradrenalin, dopamine and serotonin in the prefrontal cortex	[30]
*Behavious mouse* Elevated plus maze	1–30 mg/kg per os Active control: Lorazepam 5mg/kg Active control: Diazepam 2.5mg/kg Pregabalin 100mg/kg	3 days	Increased time in open arm: ⇒ *Effective range*: *1–30mg/kg* ⇒ *Maximum effect at 10mg/kg*	[26, 27]
*Behavious rat* Elevated plus maze Elevated zero maze Open field behaviour	3–30 mg/kg i.p. Active control: Lorazepam 5mg/kg	7 days	Increased time spend: ⇒ *in open arm* ⇒ *on open arm* ⇒ *in the open field*	[90]
Novelty-induced suppressed feeding latency			Reduced time to start feeding: ⇒ *Effective range*: *3–30mg/kg* ⇒ *Maximum effect at 30mg/kg*	
Social interaction			Increased time of social interaction: ⇒ *Effective range*: *3–30mg/kg* ⇒ *Maximum effect at 30mg/kg*	
**Antidepressant properties**				
**In vitro**				
*Rat* PC12 cells *Human* SH-SY5Y *neuroblastoma cells* Microscopy techniques	0.1, 1, 10 µg/ml Active control: NGF 50ng/ml Negative control: Pregabalin 1–30 µM	72 h	Initiation of neurite outgrowth: ⇒ *Effective range:* *0.1–10 µg/ml* GAP-43 enriched in the extending neurite: ⇒ *Effective concentration:* *10 µg/ml*	[37]
Western blot analysis	0.1, 1, 10 µg/ml	72h	Phosphorylation of protein kinase A and CREB: ⇒ *Effective concentration:* *10 µg/ml*	
*Rat* Primary hippocampal neurons Microscopy techniques	0.1, 1 µg/ml Active control: BDNF 50 ng/ml	24h	Increased number of synapses: ⇒ *Effective range:* *0.1, 1 µg/ml*	
**In vivo**				
*Freely moving rats* Microdialysis probe into the medial prefrontal cortex	3,10 or 30 mg/kg i.p.	7 days	Increased extracellular levels of noradrenalin, dopamine and serotonin in the prefrontal cortex.	[30]
*Behavious rat* Forced swimming test	1–30 mg/kg per os Active control: Imipramine 30mg/kg Negative control: Pregabalin 100mg/kg	3 days	Decreased time of immobililty: ⇒ *Effective range* *10–30 mg/kg* ⇒ *Maximum effect at 30 mg/kg*	[27, 37]
		9 days	Decreased time of immobililty: ⇒ *Effective range* *3–30 mg/kg* ⇒ *Maximum effect at 30 mg/kg*	

In anxiety disorders, certain neurons in key brain regions become hyperactivated (Fig. 1a), contributing to the heightened state of arousal, fear, and worry characteristic of these conditions. Hyperactivation refers to the excessive firing or increased excitability of neurons, often due to an imbalance between excitatory and inhibitory signals in the brain. Anxiolytic drugs encompass a range of mechanisms that reduce anxiety. Among these, gabapentin and pregabalin stand out due to their unique mechanism of inhibiting voltage-gated calcium channels (VGCCs) (Fig. 1b). The brain expresses several types of VGCCs based on their activation and inactivation properties as well as the characteristics of their pore-forming α1 subunits (Cav1-3), with the main types being the high-voltage-activated channels of the L-type (Cav1.2, Cav1.3), N-type (Cav2.2), P/Q-type (Cav2.1), R-type (Cav2.3) and the low-voltage-activated T-type calcium channel (Cav3.1, Cav3.2, Cav3.3) [25]. When an action potential reaches the presynaptic terminal, it causes VGCCs to open, allowing an influx of calcium (Fig. 1a). This rise in intracellular calcium triggers synaptic vesicles to fuse with the presynaptic membrane and release their neurotransmitter content into the synaptic cleft (Fig. 2a). The Cav1 and Cav2 families also contain the auxiliary α2δ (α2δ1, α2δ2) and β subunits (Fig. 1a). Especially the α2δ1 complex seems to represent a primary binding target for drugs like pregabalin and gabapentin. The α2δ1 subunit is a complex protein composed of two parts, which are linked by disulfide bonds and together form a large extracellular domain that is commonly associated with the P/Q-type (Fig. 1b). When pregabalin or gabapentin binds to the α2δ1 subunit, it modulates the function of the P/Q-type (Cav2.1) (Fig. 1b). Their affinity for the N-type (Cav2.2) is much lower. The modulation of the P/Q-type VGCC results in decreased calcium influx into neurons upon activation of the channels at the presynaptic terminal (Fig. 1b). This leads to a reduced release of excitatory neurotransmitters, such as glutamate and substance P, into the synaptic cleft. Consequently, there is decreased neuronal excitability and more stabilized neuronal activity at the postsynaptic terminal. The binding site for both drugs on the α2δ1 subunit of VGCCs has been identified through molecular studies. They attach to a specific site on the α2 portion of the α2δ1 subunit. The binding involves a critical arginine residue (Arg-217) located in the extracellular domain of the α2 region (Fig. 1b, upper panel). This residue seems to be essential for the high-affinity binding of pregabalin and gabapentin to the α2δ1 subunit and a critical component of their anxiolytic action.

Of note, silexan has been shown to have a similar impact on presynaptic VGCCs by inhibiting these channels, but unselectively (Figs. 1c and 2a). In contrast to pregabalin acting preferentially on the P/Q-type, silexan reduced the activity mainly of T-type and N-type channels and to some extent of P/Q-type channels in genetically modified cell models expressing specifically one of the three VGCCs [26, 27]. In each case, channel activity was reduced by about 20%. Thus, silexan seems to have a moderate effect on VGCCs (Fig. 1c), while pregabalin substantially inhibited P/Q channel function with a maximum inhibition of about 40% - twice that of silexan – leading also to a more pronounced reduction in calcium influx compared to silexan [26] (Table 1) (Fig. 1b). Hence, the moderate action of silexan on VGCCS may contribute to its favourable safety profile [28] compared to pregabalin, which exhibits a potential for tolerance and addiction that might be due to downregulation or desensitization of the α2δ1 subunit or associated calcium channels in response to chronic drug exposure [29]. While silexan has been reported to affect calcium signaling via VGCCs, potentially contributing to its anxiolytic effects, it does not specifically bind to the α2δ1 subunit (Fig. 1c) [26]. Additionally, T-type VGCCs function independently of auxiliary subunits like α2δ1, confirming that any affinity for α2δ1 does not play a role in the mechanism of action of silexan. Instead, silexan might act directly by binding and inhibiting the channel complex or indirectly by influencing calcium ion fluxes through other mechanisms not yet identified, such as modulation of surface expression of VGCC subunits (Fig. 1c), thereby normalizing the hyperexcitability of neurons involved in fear and anxiety processing (Fig. 2a).

Additionally, silexan might affect serotonin receptors (such as 5-HT1A) and modulate serotonin levels, contributing to its anxiolytic effects (Fig. 2b). Serotonin has a complex role in anxiety, affecting various brain regions and mechanisms. SSRIs work by inhibiting the reuptake of serotonin into presynaptic neurons, increasing serotonin levels in the synaptic cleft, and enhancing serotonergic neurotransmission. In line, silexan was shown to increase extracellular serotonin and dopamine in the prefrontal cortex of awake rats after a single acute administration, and this activity may contribute to the clinically observed relaxing and anxiolytic action of silexan [30] (Fig. 2b). However, unlike SSRIs, this increase in serotonin after acute exposure to silexan is not due to the inhibition of the serotonin reuptake transporter. Silexan may indirectly elevate extracellular serotonin levels in the prefrontal cortex, possibly through its effects on other brain regions.

Furthermore, a widespread reduction of the 5-HT1A receptor binding potential in the hippocampus and the anterior cingulate cortex following prolonged administration of silexan compared with placebo was detected in a clinical study [31] (Fig. 2b). The reduction is mainly based on changes in receptor expression or affinity. This is in accordance with findings after prolonged exposure of SSRIs showing a reduced 5-HT1A receptor binding as well, e.g., after 12 weeks of escitalopram intake in patients suffering from anxiety disorders in the subgenual cortex, hippocampus and posterior cingulate cortex [32]. Prolonged SSRI administration is known to cause desensitization of post-synaptic 5-HT1A heteroreceptors. Similarly, these observations suggest that prolonged treatment with silexan may induce comparable adaptive changes, such as reduced 5-HT1A receptor binding (Fig. 2b), potentially contributing to its anxiolytic effects in a manner akin to citalopram (Table 1).

Reduced 5-HT1A receptor binding may be involved in both affective and anxiety disorders, as suggested by the high comorbidity rates between these conditions and the effectiveness of SSRIs in treating both. In line, silexan has shown not only anxiolytic but also antidepressant effects (Fig. 2b). Studies in mice or rats already demonstrated that essential lavender oils and its lead ingredient linalool attenuated depressive-like behaviour [33, 34]. The antidepressant mode of action of silexan, while not fully understood, probably involves several mechanisms that influence neuroplasticity and neurotransmission (Fig. 2c). In addition to affecting serotonin neurotransmission as described in the previous paragraph, the stimulation of neuroplasticity is considered relevant for antidepressant treatment. The promotion of neuroplastic changes helps reverse the negative effects of chronic stress and depression on the brain and might represent the common final pathway of the mechanism of action of most if not all antidepressants [35, 36]. By enhancing synaptic plasticity, antidepressants improve the communication between neurons. This can lead to better mood regulation and cognitive function. In addition, antidepressants can induce changes in the structure of dendrites, the branching parts of neurons that receive synaptic inputs [35, 36]. This remodelling enhances the brain’s ability to adapt and reorganize in response to new experiences or environmental changes.

In accordance, silexan was shown to enhance several parameters of neuroplasticity within a concentration range (0.1, 1.0, 10.0 µg/ml) (Table 1), which has also been shown to be active in experiments demonstrating the inhibitory effect of silexan on VGCCs function (Fig. 2c) [37] and corresponds to plasma and tissue levels of silexan in animals and humans at therapeutically relevant doses [27, 38]. In the study by Friedland and colleagues [37], silexan demonstrated antidepressant-like effects in cellular as well as animal models for antidepressant activity (Table 1). Silexan induced neurite outgrowth (increase in neurite length) and synaptogenesis in neuronal cell models (PC12 cells, human neuroblastoma SH-SY5Y cells) and led to a significant increase in synaptogenesis in primary hippocampal neurons (increase in proteins of the pre- and post-synapse: synapsin and PSD95 resepectively) (Fig. 2c, zoomed-in inset). The cAMP-PKA pathway is a critical signalling cascade that impacts synaptic plasticity [39]. Neurotransmitters bind to G-protein-coupled receptors (GPCRs) on the cell surface and activate adenylyl cyclase which converts ATP to cyclic AMP (cAMP). cAMP binds to the regulatory subunits of Protein Kinase A (PKA). PKA translocates to the nucleus and phosphorylates the transcription factor CREB (cAMP response element-binding protein). One of the critical genes activated by CREB is the BDNF (brain-derived neurotrophic factor) gene. The cAMP-PKA-CREB-BDNF pathway plays a vital role in long-term potentiation (LTP) and long-term depression (LTD), which are cellular mechanisms underlying synaptic plasticity (Fig. 2c). Notably, silexan was shown to act via this pathway initiating phosphorylation of PKA and subsequent CREB phosphorylation (Fig. 2c) [37]. These findings support the idea that silexan might share some common mechanisms modulating synaptic plasticity with other antidepressants, particularly the SSRIs, with fluoxetine considered the gold standard [38]. In line with these findings, another study demonstrated that essential lavender oil (daily inhalation exposure for 14 days) not only improved depression-like behaviour in rats (depression- and anxiety-like behaviour was induced by high corticosterone administration) but also promoted neurogenesis and improved dendritic branching in the hippocampus of the treated rats [40]. In contrast, the anxiolytic drug pregabalin was devoid of any antidepressant mode of action in vitro as well as in vivo [37] suggesting that its inhibitory effects on VGCCs may not contribute to antidepressant mechanisms. However, it is possible that silexan’s effects on intracellular signaling molecules within the cAMP-PKA-CREB pathway not only enhance neuroplasticity but also impair various VGCCs. Together, these mechanisms may contribute to the beneficial effects of the drug on anxiety and depressive disorders [38].

## Clinical trials in anxiety disorders

The anxiolytic effect of silexan was assessed in 6 randomized, double-blind, placebo and/or reference controlled clinical trials. One study investigated patients with subthreshold anxiety disorder coded as ‘Anxiety disorder not otherwise specified’ (ICD-10 F41.9, DSM-IV 300.00) [41], one included patients suffering from restlessness, agitation and disturbed sleep (ICD-10 R45.1) secondary to anxious moods [42], and one was performed in patients with mixed anxiety and depressive disorder (MADD; ICD-10 F41.2) [43]. Three studies in patients with GAD compared silexan to lorazepam [44], to placebo and paroxetine [45], and to placebo alone [46]. Reference-controlled studies used a double-dummy design. The absolute change of the total score of the Hamilton Rating Scale for Anxiety (HAMA) [47] between baseline and treatment end was pre-defined as the primary efficacy outcome measure in all studies. At baseline, average HAMA total scores ranged between 24.6 and 27.1 points. Table 2 provides a summary of further trial characteristics.

**Table 2:** Clinical trials investigating the anxiolytic efficacy of silexan

Trial	Inclusion diagnosis	Interventions^a^	HAMA total score decrease (points), silexan 80 mg/d – placebo^b^
A: Kasper et al., 2010 [41]	Anxiety disorder not otherwise specified	80 mg/day silexan (*n* = 104) or placebo (*n* = 108), 10 weeks	6.5 (4.1; 8.8), *p* < 0.01
B: Kasper et al., 2015 [42]	Restlessness, agitation, and disturbed sleep	80 mg/day silexan (*n* = 86), or placebo (*n* = 84), 10 weeks	2.7 (0.3; 5.0), *p* = 0.03
C: Kasper et al., 2016 [43]	Mixed anxiety and depressive disorder	80 mg silexan (*n* = 159) or placebo (*n* = 156), 10 weeks	2.5 (0.5; 4.5); *p* < 0.01
D: Woelk and Schläfke, 2010 [44]	Generalized anxiety disorder	80 mg/day silexan (*n* = 40) or 0.5 mg/ day lorazepam (*n* = 37), 6 weeks	Not applicable
E: Kasper et al.; 2014 [45]	Generalized anxiety disorder	80 mg/day silexan (*n* = 135), 20 mg/day paroxetine (*n* = 132), or placebo (*n* = 135), 10 weeks	2.8 (0.9; 4.8); *p* < 0.01
F: Kasper et al., 2017 [46]	Generalized anxiety disorder	80 mg silexan (*n* = 103) or placebo (*n* = 102), 10 weeks	0.5 (-1.7; 2.6); *p* = 0.16

^a^ Number of patients: full analysis set

^b^ Full analysis set, last observation carried forward. Data are baseline-adjusted mean value differences and their 95% confidence intervals from analysis of variance models. Positive values favour silexan

The results presented in this section pertain to the authorized posology of silexan, which is 80 mg once daily, and to the full analysis sets (FAS), unless otherwise stated. Trial E [45] also included a patient group that took 160 mg daily and trial F included groups with 10 or 40 mg daily [46]. The results for these groups have been reported elsewhere [45, 46].

**Fig. 3:**
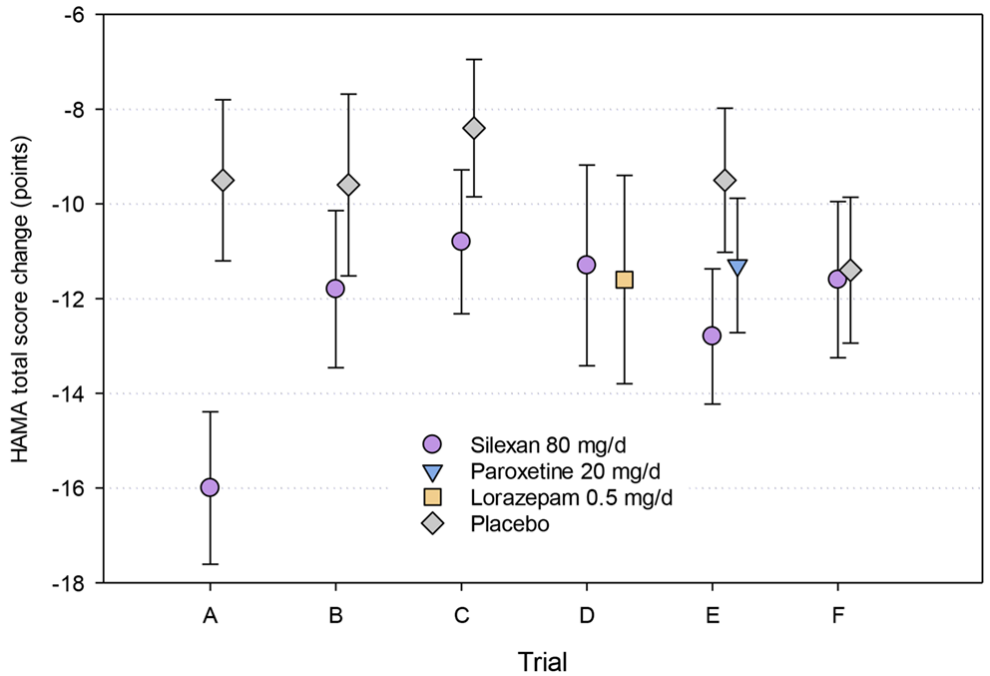
Hamilton Anxiety Rating Scale (HAMA) total score change between baseline and treatment end (means and 95% confidence intervals; full analysis set; last observation carried forward; for sample sizes, see Table 2)

In patients with subthreshold anxiety (including MADD), silexan was significantly superior to placebo in reducing the HAMA total score between baseline and end of treatment in all 3 available trials (A–C; Fig. 3; Table 2).

In syndromal GAD, Trial D, which was performed as a proof-of-concept study, showed equal HAMA total score reductions for 80 mg/d silexan and lorazepam administered at the initial therapeutic dose of 0.5 mg/d (90% confidence interval [CI] for the treatment group difference: -2.7; 2.4 points) after 6 weeks. However, the proportion of responders (patients with a HAMA total score reduction by at least 50% compared to baseline) as well as the proportion of patients in remission (HAMA total score < 10) at treatment end were significantly larger in the silexan group compared to lorazepam (52.5% vs. 40.5%, *p* = 0.04, and 40% vs. 27, *p* = 0.04, respectively). In study E, the HAMA total score reduction in the silexan 80 mg/d group was significantly greater than in the placebo group and at least as great as in patients treated with 20 mg/d paroxetine. Patients in the dosage group 160 mg silexan once daily showed even more pronounced HAMA total score improvements than those in the 80 mg/d group, with no clinically meaningful increase in adverse events (see [45] for details).

Study F was a dose-ranging trial performed to identify the lowest effective dose of silexan in GAD. Significant differences to placebo could, however, not be shown, due to unexpectedly large HAMA total score decreases in the placebo group that was in the range of those observed for the active drugs in the other trials (Fig. 3).

A meta-analysis was performed of all placebo-controlled trials with silexan in anxiety disorders [48]. For the HAMA total score reduction in Trials A-C, E and F, the authors determined a meta-analysis mean value difference favouring silexan 80 mg/d and of 2.93 points (95% CI: 1.07; 4.77 points) compared to placebo (*p* < 0.001). In the same meta-analysis, superiority of the lavender oil over placebo was somewhat more pronounced for the psychic anxiety subscale of the HAMA as compared to the somatic anxiety subscale even though both showed significant treatment group differences. On an individual-item basis, treatment effects were most pronounced for anxious mood, tension, and insomnia, followed by intellectual impairment, depressed mood and by somatic sensory and muscular impairment. The meta-analysis resulted significant differences between silexan and placebo for all items. Based on HAMA total score change, the responder rates across all trials were 51.8% and 38.8% for silexan and placebo, respectively [48].

The results for the HAMA were fully supported by the secondary efficacy outcomes used in the trials. For different anxiety self-rating scales (Trials A, B: Zung Self-Rating Anxiety Scale [49]; Trial C: Covi Anxiety Scale [50]; Trials E, F: Hospital Anxiety and Depression Scale [51]), a standardized mean value difference between silexan and placebo of 0.27 (95% CI: 0.15; 0.38) standard deviation units was observed favouring silexan (*p* < 0.001). The results were also reflected in the investigators’ ratings in the Clinical Global Impressions (CGI [52] scale where silexan-treated patients were on average 51% more likely to be much or very much improved than placebo-treated patients according to the risk ratio (*p* < 0.001).

Based on the data from Trials A–C, E and F the effects of silexan on somatic symptoms, physical health, and health-related quality of life were summarised [53]. Analyses were based on the items of the somatic anxiety subscale of the HAMA as well as on the SF-36 health status questionnaire [54] that was administered in Trials A, C, E and F. In the HAMA subscale, a standardized mean difference of 0.31 (95% Cl: 0.10; 0.52) favouring silexan was observed in a meta-analysis (*p* < 0.01). Treatment effects of silexan on somatic anxiety were independent of sex and age. Similar clinically meaningful effects of silexan were demonstrated for SF-36 physical health, including reduced bodily pain and improved general health, and on insomnia complaints and fatigue.

## Clinical trials in depression

**Fig. 4:**
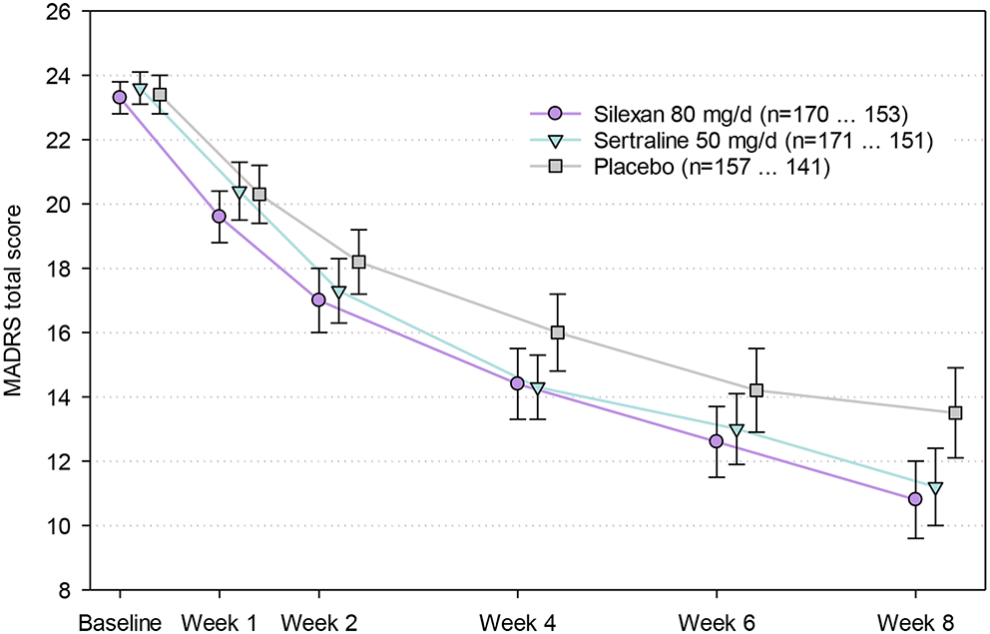
Montgomery-Åsberg Depression Rating Scale (MADRS) total score time courses (means and 95% confidence intervals; full analysis set; primary treatment policy estimand without imputation of missing data; legend shows numbers of subjects with valid data at baseline and week 8). From [55]

A recently published, confirmatory trial investigated the antidepressant effect of silexan in patients with mild to moderate MDD, single or recurrent episode (ICD-10 F32.0, F32.1, F33.0, or F33.1) [55]. Participants were randomized to take 80 mg/d silexan, 50 mg/d sertraline, or placebo double-blind for 8 weeks. The primary outcome measure for efficacy was the absolute change of the Montgomery-Åsberg Depression Rating Scale (MADRS) [56] between baseline and treatment end using an estimand framework to account for missing or invalid data. 498 subjects (silexan 170, sertraline 171, placebo 157) were treated and analysed. Figure 4 shows the MADRS total score time courses. After 8 weeks, silexan and sertraline were superior to placebo for MADRS total score reduction, with statistically significant differences to placebo of 2.17 (95% CI: 0.58; 3.76) and 2.59 (1.02; 4.17) points, respectively (*p* < 0.01; baseline- und centre-adjusted mean value differences for treatment policy estimand with multiple missing data imputation). Moreover, silexan was superior to placebo for alleviation of MDD-associated functional impairment according to the Sheehan Disability Scale [57], with a total difference of 2.40 (95% CI: 1.04; 3.76) points (*p* < 0.01) and significant differences to placebo for each of the sub-domains of the scale (work, social life/leisure activities, family life/home responsibilities; *p* < 0.01) whereas no significant improvements over placebo were observed for sertraline. The study thus confirmed the antidepressant efficacy of silexan in patients with mild or moderate MDD [55].

**Fig. 5:**
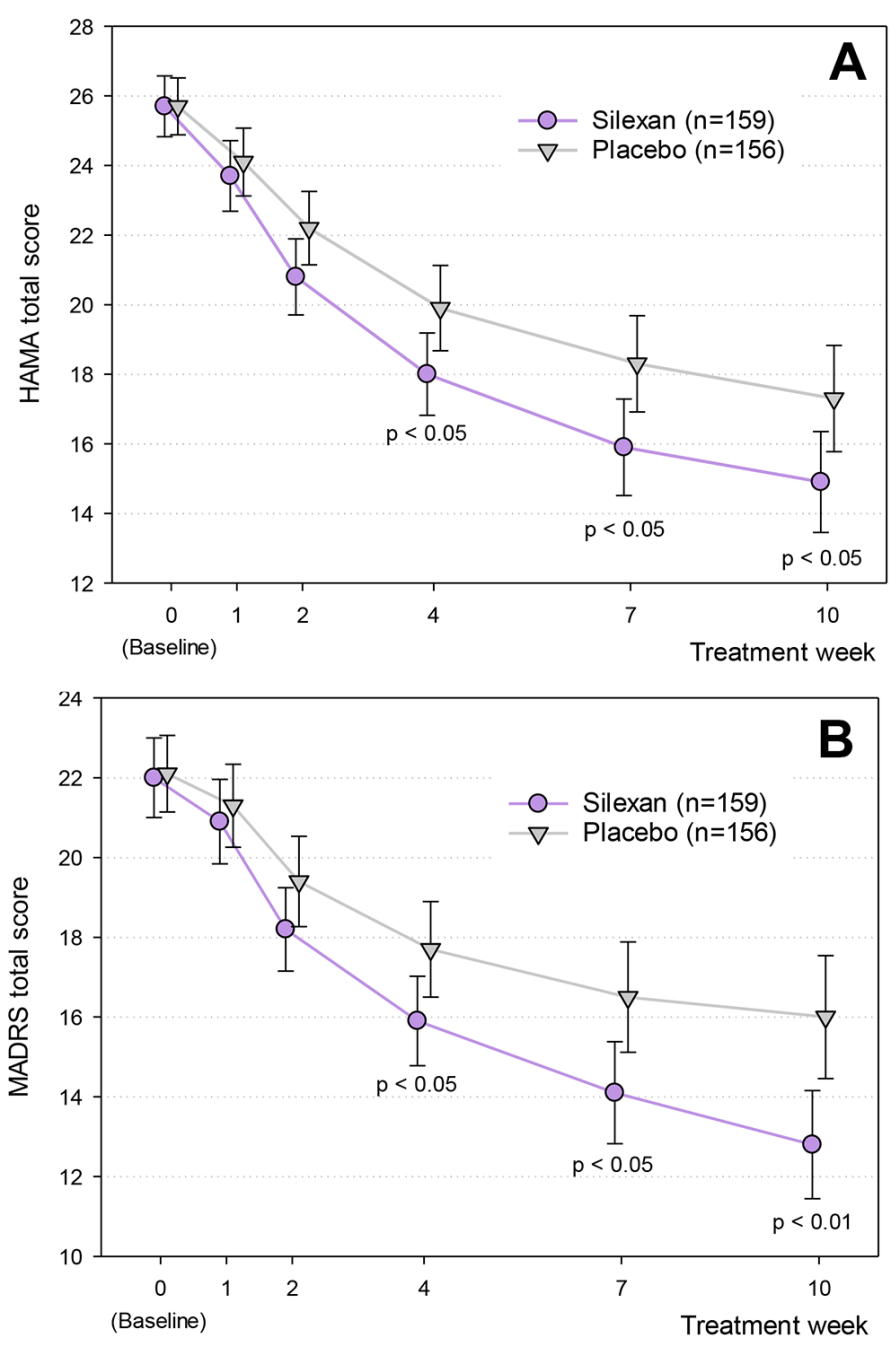
Hamilton Anxiety Rating Scale (HAMA, Panel **A**) and Montgomery-Åsberg Depression Rating Scale (MADRS, Panel **B**) time course (means and 95% confidence intervals, full analysis set, last observation carried forward). Adapted from [43]

MADD (ICD-10 F41.2) is characterized by subsyndromal symptoms of anxiety and depression, neither of which are clearly predominant. A double-blind, randomized, placebo-controlled study [43] in Table 2 and in Fig. 4 as trial C demonstrated that in patients with MADD, 80 mg/d silexan has a beneficial effect on both symptoms of anxiety and depression. Using absolute MADRS and HAMA total score change between baseline and week 10 as co-primary outcome measures, statistically significant effects were observed already after 4 weeks of treatment, and the differences to placebo increased in magnitude until treatment end (Fig. 5). At week 10, analysis of covariance showed average MADRS total score changes (adjusted for centre and the MADRS baseline value) of -9.2 ± 9.9 and − 6.1 ± 7.6 points for silexan and placebo, respectively (means ± standard deviations) and an adjusted mean value difference of 3.25 (95% Cl: 1.36; 5.14) points favouring silexan (*p* < 0.01). Compared to placebo, the patients treated with silexan also had a better over-all clinical outcome according to the CGI and showed more pronounced improvements of impaired activities of daily living and health related quality of life in the Sheehan Disability Scale and in the SF-36.

The HAMA includes an item for assessing depressed mood, the ratings of which are thus available for all trials listed in Table 2. Moreover, the test battery administered in Trials E and F also included the Hamilton Rating Scale for Depression (HAMD) [58] while trial C included the MADRS for the assessment of depression. In a meta-analysis of trials A–C, E and F based on HAMA item ‘Depressed Mood’, Bartova and colleagues [59] found a more pronounced antidepressant effect for silexan as compared to placebo in 4 of the 5 studies, with a meta-analysis mean value difference of 0.21 (95% CI: 0.04; 0,38) points (*p* < 0.01; full analysis set). In a subset of patients with at least moderately severe depressed mood at baseline, the meta-analysis treatment group difference favouring silexan was even more pronounced (0.27 [95% CI: 0.07; 0.47] points; *p* < 0.01). The results are supported by a meta-analysis of the specific anxiety rating scales used in trials C, E and F for which silexan was superior to placebo in reducing the symptoms of depression by a standardized mean value difference of 0.30 (95% CI: 0.16; 0.44) points for all patients in the full analysis set (*p* < 0.01) and of 0.33 (95% CI: 0.18; 0.48) points for patients with at least mild depression at baseline (*p* < 0.01). The analysis also revealed that patients with SAD (trials A and B) tended to exhibit more severe symptoms of depression at baseline than those with syndromal GAD (trials E and F).

## Clinical effects on sleep quality

Patients with anxiety disorders often suffer from insomnia, including delayed onset, disrupted sleep quality, and increased or early awakenings [60]. Vice versa, poor sleep adversely affects the symptoms of anxiety [61] so that insomnia is an important target of anxiolytic treatment [62]. Seifritz and colleagues [63] reviewed 3 studies (trials A, B, D) in which insomnia was assessed using specific, validated scales. Moreover, they performed a meta-analysis of HAMA item Insomnia based on studies A–C, E and F. Silexan was superior to placebo in reducing insomnia by a mean value difference of 0.32 (95% CI: 0.10; 0.54) points (*p* < 0.01). The authors further observed beneficial effects on sleep latency, duration, and daytime sleepiness.

In the pharmacological treatment of anxiety and impaired sleep with drugs such as SSRIs, SNRIs, benzodiazepines or antihistamines, improvements of insomnia are at least partly attributable to a direct sedating effect of the medication. A mediation analysis based on the data of trial A showed, however, that the direct effect of silexan on sleep impairment is negligible. Instead the beneficial effect of the active ingredient on sleep is mediated by its anxiolytic action [64].

An analysis of prescription data [65] revealed that the administration of silexan for disturbed sleep (ICD-10: G47.0 or G47.9) was associated with a significantly lower repeat consultation rate for the same disorder than treatment with benzodiazepine receptor agonists (zolpidem, zopiclone, and zaleplon; odds ratio: 0.56; 95% CI: [0.51; 0.60]).

## Silexan for anxiety and depressive disorders in post-COVID

The emergence of the coronavirus-19 disease (COVID-19) pandemic in 2020 was associated with a global increase in the prevalence of anxiety and depressive disorders [2, 66]. A series of case reports published by Bartova and colleagues [67] provides first evidence that patients suffering from subsyndromal anxiety and depression, MADD, or GAD in the context of post-COVID-19 syndrome may benefit from treatment with silexan.

As outlined in the previous sections, subsyndromal anxiety and depression, which are particularly common in patients with post-COVID 19 syndrome, have been shown to benefit from treatment with silexan. The therapeutic profile of the herbal medicinal product thus matches well with the psychiatric manifestations of post-COVID 19 syndrome [68].

## Tolerability in clinical trials

Eructation and gastrointestinal complaints such as nausea and dyspepsia were adverse events that occurred more frequently with silexan than with placebo in clinical trials [41, 43–46, 55]. The risk of gastrointestinal events was estimated to be about 3% higher than with placebo [69]. Silexan has not been shown to cause sexual dysfunction, weight gain or sleep disturbance, which are common adverse effects of psychiatric drugs. The lavender oil has no sedative effect [64] and does not impair the ability to drive or operate machinery [70]. It has no measurable potential for abuse and does not cause withdrawal effects [71]. It was demonstrated that silexan does not interact with the cytochrome P450 enzyme system, and thus does not have the potential for pharmacokinetic drug interactions [72]. The postmarketing experience has not yielded any evidence of pharmacodynamic drug interactions. In particular, there is no influence on the efficacy of hormonal contraceptives [73], which is important given the relatively high prevalence of anxiety and depression in young women [74]. In patients with severe renal impairment (Creatinine-Clearance < 30 ml/min/1,73 m^2^) silexan was well tolerated and linalool plasma levels did not indicate a need for dose-adjustment [75].

## Discussion and conclusions

The current clinical guidelines of the World Federation of Societies of Biological Psychiatry (WFSBP) and of the Canadian Network for Mood and Anxiety Treatments (CANMAT) taskforce for the treatment of psychiatric disorders with nutraceuticals and phytopharmaceuticals include a provisional recommendation for *Lavandula officinalis L.* for the treatment of GAD, and a weak recommendation for the treatment of MDD. Both recommendations apply to monotherapy and adjuvant treatment. The guideline committee advises that standardised essential oil is potentially more effective than dried plant material and suggests a daily dose of 80 mg–160 mg per day of a specialised oil (in capsule form) [76]. It should be noted that the anxiolytic and antidepressant properties of silexan have thus been recognized by professional organizations even without considering the clinical trials performed in subthreshold presentations of anxiety and depression (trials A–C) [41–43] and before the first trial confirming the antidepressant efficacy of silexan in MDD [55] was published.

This review is based on data from all double-blind, randomised, placebo and reference-controlled clinical trials with silexan in anxiety and depression published by June 2024. The results underline that silexan has a clinically relevant anxiolytic effect in both subthreshold and syndromal anxiety disorders. The meta-analysis published by Möller and colleagues [77] shows that the magnitude of the anxiolytic effect of silexan was in the range of that reported for synthetic drugs recommended by disease management guidelines as first-line treatment for anxiety disorders. Moreover, the herbal medicinal product was demonstrated to exert significant antidepressant effects in patients with MDD, MADD, and depression accompanying an anxiety disorder. Importantly for the concept of rational phytotherapy, the anxiolytic and antidepressant effects of silexan are plausible based on the findings of pharmacodynamic studies [78, 79].

In clinical practice, subthreshold anxiety and depression are far more prevalent than syndromal presentations [80–82]. Even though patients with SAD or SDD do not meet al.l criteria of GAD and MDD, respectively, their disease-specific symptom scores may be in the same range as those of patients with syndromal diagnoses, as shown for SAD vs. MAD in the meta-analysis of Dold and colleagues [48]. They are thus affected by comparable co-morbidities, experience similar adverse effects on their quality of life, and are at an increased risk of an exacerbation to a fully syndromal presentation of their disorder.

In particular, in cases of mild or subthreshold anxiety and depression, anticipated side effects of synthetic drugs may act as a deterrent for patients to pursue pharmacotherapy or for doctors to prescribe it [83]. This may result in patients remaining untreated, even though they are suffering and in need of treatment [84]. It is therefore essential that an effective medication with a low side effect profile is made available to meet their medical needs.

Although anxiety and depressive disorders are more prevalent in females than in males, there has been a paucity of research investigating the potential consequences of sex and gender on the treatment of these conditions [85]. Given the relatively high prevalence of anxiety and depression in younger women [74, 86], it is reassuring that no indications for interactions between silexan and oral contraceptives have been observed [73].

Clinical studies and the postmarketing experience have not yielded any evidence of interactions of silexan with other drugs. This is important in elderly patients where depression and anxiety are particularly prevalent [87]. They are a highly vulnerable group often with multiple chronic complaints where polypharmacy is the rule rather than the exception. The high potential for often complex drug interactions makes treatment often challenging. Moreover, side effects such as fatigue, sedation, somnolence, which have been described even for newer-generation chemical antidepressants and anxiolytics [21, 88], may further diminish the patients’ already impaired functional level and may, for example, increase the risk of falls and cognitive impairment. A review and meta-analysis [89] shows that silexan is safe and effective in the elderly population.

In conclusion, based on the results of 7 randomized, double-blind, placebo and reference-controlled clinical trials, silexan was demonstrated to be safe and effective in subthreshold and syndromal anxiety and depressive disorders. It may further be a promising option to improve sleep quality and treat anxiety or depressive disorders in individuals with post-COVID-19. The clinical effects of silexan are consistent with its pharmacological profile. Nevertheless, further studies are necessary to understand better the efficacy of silexan across various anxiety and depression disorders and its comparative effectiveness against established pharmacological treatments. Additionally, more research is needed to clarify the precise mechanisms of action of silexan at the molecular and cellular levels, including its effects on serotonergic neurotransmission, receptor pathways, and the modulation of VGCCs. These investigations could pave the way for developing more targeted therapies and expand the therapeutic applications of silexan to a wider range of conditions.

## Data Availability

All the supporting data and information are available within the article.

## Footnotes

1 Silexan^®^ is the active substance of Lasea^®^ (Dr. Willmar Schwabe GmbH & Co. KG, Karlsruhe, Germany).
